# Understanding who benefits most from cognitive rehabilitation for multiple sclerosis: A secondary data analysis

**DOI:** 10.1177/13524585231189470

**Published:** 2023-08-01

**Authors:** LA Taylor, JR Mhizha-Murira, G Law, N Evangelou, R das Nair

**Affiliations:** Mental Health and Clinical Neurosciences Unit, School of Medicine, Institute of Mental Health, University of Nottingham, Nottingham, UK; College of Social Science, University of Lincoln, Lincoln, UK; Faculty of Medicine and Health Sciences, University of Nottingham, Nottingham, UK; Faculty of Medicine and Health Sciences, University of Nottingham, Nottingham, UK; SINTEF Digital, Trondheim, Norway

**Keywords:** Multiple sclerosis, neurological rehabilitation, neuropsychology, cognition, memory, attention

## Abstract

**Background:**

Up to 70% of people with multiple sclerosis (MS) experience cognitive difficulties. Cognitive rehabilitation is a type of therapy that helps manage cognitive problems.

**Objective:**

The Cognitive Rehabilitation for Attention and Memory in MS (CRAMMS) trial showed some evidence of effectiveness of cognitive rehabilitation in improving cognitive function, with some participants benefitting more than others. We therefore conducted a secondary analysis of the CRAMMS data to understand who benefits most.

**Methods:**

We grouped baseline data into four categories of possible predictors. We used regression models to identify specific factors/characteristics that could predict the likelihood that an individual will benefit from cognitive rehabilitation.

**Results:**

The models predicted whether a participant improved or did not improve in neuropsychological function following cognitive rehabilitation in up to 86% of participants. Results suggest that younger participants with medium to high education, diagnosed with relapsing-remitting multiple sclerosis (RRMS) and primary-progressive multiple sclerosis (PPMS) who have not experienced any recent relapses, with mild to moderate cognitive difficulties were most likely to benefit from cognitive rehabilitation.

**Conclusion:**

We can predict which participants are most likely to demonstrate significant improvements in neuropsychological function following group-based cognitive rehabilitation. Clinically, this allows us to optimise limited neuropsychology resources by offering such cognitive rehabilitation to those most likely to benefit.

## Introduction

Cognitive difficulties with memory, attention, planning, speed of information processing, and problem-solving affect up to 70% of people with multiple sclerosis (MS),^1^ which can interfere with people’s ability to complete everyday tasks, creating distress for those with MS and their families.^2^ Cognitive rehabilitation provides people with MS with the knowledge of and information about their cognitive problems, and teaches them to use internal and external aids to address them.^3,4^ Attention and memory rehabilitation are major components of this type of intervention and are important because they are common cognitive difficulties reported in MS.^5^ Attention rehabilitation teaches people better ways of paying attention, reducing distraction, and improving concentration. Memory rehabilitation teaches different strategies for encoding, storing, and retrieving memories and is suggested to involve targeted, repeated stimulation to certain brain areas, thought to trigger the activation of neural networks.^6,7^ For group-based interventions, the therapeutic effects of being with others with similar problems may also help participants engage with the rehabilitation and subsequently lead to greater improvements in cognitive function and psychological well-being.^8,9^

Although studies have yielded mixed evidence regarding the effectiveness of this intervention,^10–12^ a recent systematic review found evidence in support of cognitive rehabilitation for MS.^13^ However, the degree to which patients benefit from cognitive rehabilitation appears to vary. This could be because of extremely broad inclusion criteria in terms of socio-demographics and clinical characteristics in research studies. Therefore, there is a need to determine which subgroups of people with MS benefit most from cognitive rehabilitation because this will help clinicians decide to whom this intervention is offered.

Clinical prediction tools help make such decisions, matching patients to treatments to yield the optimum outcomes/benefits, in line with the principles of precision medicine.^14^ This (1) helps optimise Health Services resources in an efficient way (currently, very few centres offer cognitive rehabilitation due to limited number of clinicians available to offer the intervention) and (2) avoids offering unnecessary interventions to those unlikely to benefit (reducing unnecessary travel to the therapy sessions and any frustration of having participated in a treatment that does not benefit patients). There appears to be no developed clinical prediction tools to determine who would benefit most from group-based cognitive rehabilitation. We therefore aimed to close this research gap within this study.

The primary aim of this study was to determine which groups of people with MS improved more in cognition and psychological well-being following cognitive rehabilitation.

## Methods

### Study sample and design

This is a secondary data analysis of the Cognitive Rehabilitation for Attention and Memory in Multiple Sclerosis (CRAMMS) trial,^15^ the largest Phase III randomised controlled trial that evaluated the clinical and cost-effectiveness of a group-based cognitive rehabilitation programme for people with MS, involving 449 patients. The CRAMMS intervention is a group-based cognitive rehabilitation programme which focuses on teaching people with MS compensatory strategies to help them manage their memory and attention difficulties. People with MS experiencing cognitive difficulties are invited to attend 10 sessions over 10 consecutive weeks in small groups of 4–6 people, each session lasts one and a half hours. For a detailed description of this trial, please see CRAMMS monograph.^15^

### Statistical analysis

For the current analyses, we explored all outcome measures in which improvement was observed in the CRAMMS trial.

Directed acyclic graphs (DAGs) were built to determine potential confounding factors for individual regression analyses. A separate DAG was used for each dependent variable and all possible predictors. The DAGs were used to identify which predictors could potentially influence whether an individual is likely to improve in each dependent variable. These predictions were then included in the logistic regression models which were conducted using Stata. To assess for multicollinearity, we used variance inflation factor (VIF) values, ensuring that all variables included in the models were below five. Akaike information criterion (AIC) was used to ensure the final model was the best fit for the data. We calculated Nagelkerke’s pseudo *R*^2^, which indicates the explanatory power of the model (i.e. how much of the total variability of the dependent variable is explained by the model). This value ranges from 0 to 1, with scores of 0.75, 0.5, and 0.25 indicating substantial, moderate, and weak explanatory power of the model, respectively.^16^

To explore the presence of potential predictors of change in the outcome measures, univariate descriptive statistics of baseline demographics, clinical variables, self-report cognitive symptoms, objective neuropsychological test performance, and treatment dose (Table 1) were used to create graphs to identify any interesting breaks in the distributions, including very high or very low values. This also allowed us to understand how each potential predictor related on its own, to each outcome measure, and other potential predictors.

**Table 1.** Primary categories of possible predictors.

Predictor category	Predictors
Socio-demographics	Age
	Gender
	Education
	Ethnicity
	Employment status
	Relationship status
Clinical variables	Number of years a person has been diagnosed
	Type of MS
	Number of relapses 6 months prior to initial assessment
	Number of relapses within 6 months of starting the intervention
Self-reported cognitive symptoms	Everyday Memory Questionnaire-participant version (EMQ-p)
	Everyday Memory Questionnaire-relative version (EMQ-r)
	30-item General Health Questionnaire (GHQ-30)
	Multiple Sclerosis Impact Scale – Psychological subscale (MSIS-Psy)
	European Quality of Life five-level version (EQ-5D-5L)
Objective neuropsychological test performance	Brief Repeatable Battery of Neuropsychological tests (BRBN)
	Doors and people test

MS: multiple sclerosis.

The dependent variables were the difference in scores of self-report cognitive measures and neuropsychological tests between baseline and 6 month follow-up (Table 2). We used this to determine whether a participant improved or not. Each dependent variable was dichotomised to include participants who improved and did not improve following cognitive rehabilitation. Improvement was defined as positive change that occurred in scores at 6 month follow-up, when compared to their baseline scores. The direction of this change varied depending on the outcome measures, as indicated in Table 2. From the final models, we were able to understand which predictors contributed significantly to the improvement in each cognitive area following cognitive rehabilitation.

**Table 2.** Neuropsychological and subjective cognitive measures.

Neuropsychological tests		
BRBN	Brief Repeatable Battery of Neuropsychological test (1)^a^	A cognitive screening battery that mainly assess memory and attention and takes motor into account
	Symbol Digit Modalities Test	Sustained and divided attention and information processing.
	Selective Reminding Test Total recall (0–72) Delayed recall (0–12)	Verbal learning and memory
	10/36 Spatial Recall Total correct (0–30)	Visuospatial memory
	Paced Auditory Serial Addition Test (PASAT) Easy total correct Hard total correct	Auditory information processing speed and working memory
Doors and people test	Overall age-scaled score^a^ Verbal total score^a^ Non-verbal total score^a^ Total forgetting score^a^	Visual and verbal recall and recognition
Trail Making test	Part B – Part A (0–20)^a^	Visual attention and task switching
Subjective cognitive measures (questionnaires)		
EMQ-p	Everyday Memory Questionnaire- participant version^b^ (0–112)	Everyday memory
EMQ-r	Everyday Memory Questionnaire-relative version^b^ (0–112)	
MSIS-Psy	Multiple Sclerosis Impact Scale-Psychological subscale^c^	Psychological well-being
GHQ-30	30-item General Health Questionnaire^d^ (0–90)	General health over the past few weeks

a Higher scores indicate better cognitive function.

b Higher scores indicate more frequent memory problems.

c Higher scores indicate a greater negative impact of MS.

d Higher scores indicate higher levels of psychological distress.

The analyses were performed on the available data, that is, if values were missing, no imputation of missing data such as intent-to-treat was performed.

## Results

The exact number of participants included in each model (out of the 245 participants who were randomised and received the cognitive rehabilitation) varied depending on how many people completed the 6-month follow-up outcome measures. A substantial number of participants improved in cognition and psychological well-being outcomes following cognitive rehabilitation (Table 3).

**Table 3.** Frequency of improvement after cognitive rehabilitation for people with MS.

	Frequency	%	*N* total
EMQ-p			245
Improved	174	71.02	
Not improved	71	28.98	
MSIS-Psy			245
Improved	195	79.78	
Not improved	50	20.22	
SDMT			245
Improved	187	76.33	
Not improved	58	23.67	
PASAT3			245
Improved	169	68.98	
Not improved	76	31.02	
PASAT2			245
Improved	186	75.92	
Not improved	59	24.08	
S10/36			245
Improved	156	63.67	
Not improved	89	36.33	

MS: multiple sclerosis; EMQ-p: Everyday Memory Questionnaire – Participant; MSIS-Psy: Multiple Sclerosis Impact Scale – Psychological; SDMT: Symbol Digit Modalities Test; PASAT3: Paced Auditory Serial Addition Test–3 second; PASAT2: Paced Auditory Serial Addition Test – 2 second; S10/36: 10/36 Spatial Recall Test.

### Subjective memory

The final model (model *χ*^2^(9) = 168.665, *p* < 0.001) that predicted improvement in Subjective Memory following cognitive rehabilitation included predictors from all categories (Table 4). A Nagelkerke’s pseudo *R*^2^ of 0.580 indicates a moderate explanatory power of the model. The final model correctly classified 79.8% of participants. The odds of improvement were 2.16 times greater for those ‘living with others’ compared to those living alone; however, this was not statistically significant (*p* = 0.098). The odds of improvement increased as age decreased (*p* = 0.606) and education increased (*p* = 0.210); however, this was not significant.

**Table 4.** Logistic regression models.

Model	χ^2^	*df*	*p*	R^2^ (Nagelkerke)	Variables in the model	B	*SE*	Wald	*df*	Sig.	Exp(B)	95% CI for Exp(B)	
												lower	upper
Improvement in Subjective memory (EMQ-p)	168.665	9	<0.001	0.580	Age	–0.010	0.018	0.267	1	0.606	0.910	0.974	1.047
					Education	0.071	0.056	1.573	1	0.210	0.932	0.834	1.041
					Living with others	0.772	0.466	2.737	1	0.098	2.163	0.867	5.397
					Time since diagnosis	–0.035	0.023	2.369	1	0.124	0.965	0.923	1.010
					Relapses	0.583	0.370	2.484	1	0.115	1.791	0.868	3.696
					EMQ-p	–0.031	0.009	11.009	1	<0.001	0.969	0.951	0.987
					MSIS-Psy	0.000	0.033	0.000	1	0.995	1.000	0.937	1.068
					SDMT	–0.006	0.015	0.159	1	0.690	0.994	0.966	1.023
					Doors and people test								
					tfs								
					Constant	–0.187 24.814	0.052 4774.872	13.021 0.000	1 1	<0.001 0.996	0.829 5.977E+10	0.749	0.918
Improvement in psychological well-being (MSIS-Psy)	108.523	6	<0.001	0.513	Living with others	0.303	0.460	0.433	1	0.511	1.354	0.549	3.337
					Time since diagnosis	–0.031	0.026	1.395	1	0.238	0.970	0.922	1.020
					Relapses	0.760	0.442	2.957	1	0.086	2.139	0.899	5.089
					MS Type			6.359	2	0.042			
					RRMS	0.016	0.454	0.001	1	0.972	1.016	0.418	2.471
					PPMS	–1.842	0.798	5.331	1	0.021	0.158	0.033	0.757
					MSIS-Psy	0.096	0.033	7.901	1	0.005	1.101	1.029	1.177
					Constant	–17.501	3270.966	0.000	1	0.996	0.000		
Improvement in Sustained attention and Processing Speed (SDMT)	70.322	12	<0.001	0.391	Age	–0.087	0.026	11.535	1	<0.001	0.917	0.872	0.964
					Education	0.090	0.061	2.213	1	0.137	1.094	0.972	1.233
					Relapses	–0.022	0.471	0.002	1	0.963	0.979	0.389	2.462
					MS Type			4.490	2	0.106			
					RRMS	0.962	0.465	4.276	1	0.039	2.617	1.052	6.515
					PPMS	0.805	0.742	1.177	1	0.278	2.237	0.522	9.577
					MSIS-Psy	0.093	0.036	6.535	1	0.011	1.097	1.022	1.178
					Doors and people test								
					Oass								
					Vrecalls	0.779	0.425	3.367	1	0.067	2.180	.948	5.012
					Nvrcogs	0.266	0.115	5.364	1	0.021	1.304	1.042	1.633
					Vts	0.155	0.102	2.317	1	0.128	1.168	0.956	1.427
					nvts	–0.569	0.272	4.388	1	0.036	0.566	0.332	0.964
					SRT-s	–0.336	0.279	1.446	1	0.229	0.715	0.413	1.236
					SDMT	–0.028	0.016	3.007	1	0.083	0.973	0.942	1.004
					Constant	–0.132 5.951	0.025 2.523	28.240 5.565	1 1	<0.001 0.018	0.876 384.263	0.834	0.920
Improvement in Attention, vigilance and Working Memory (PASAT)	76.847	14	<0.001	0.420	Age	–0.010	0.024	0.183	1	0.669	0.990	0.943	1.038
					Education	0.014	0.058	0.055	1	0.815	1.014	0.904	1.136
					Relapses	–0.190	0.455	0.175	1	0.676	0.827	0.339	2.016
					Time since diagnosis	–0.016	0.028	0.303	1	0.582	0.985	0.932	1.041
					MS Type			1.966	2	0.374			
					RRMS	–0.590	0.506	1.358	1	0.244	0.554	0.205	1.495
					PPMS	0.950	0.792	1.438	1	0.230	0.387	0.082	1.826
					MSIS-Psy	–0.039	0.043	0.815	1	0.367	0.962	0.885	1.046
					EMQ-p	–0.012	0.012	0.985	1	0.321	0.988	0.966	1.011
					Doors and people test								
					Oass								
					Nvrecall	0.096	0.016	0.844	1	0.358	1.101	0.897	1.352
					Nvrcogs	–1.084	0.409	7.026	1	0.008	.338	0.152	0.754
					Nvts	–1.297	0.434	8.943	1	0.003	.273	0.117	0.640
					Pasat2	1.775	0.681	6.792	1	0.009	5.899	1.553	22.411
					SRT-s	–0.113	0.025	20.264	1	<0.001	0.893	0.851	0.938
					Constant	–0.012 9.469	0.016 2.893	0.557 10.713	1 1	0.456 0.001	0.988 12,954.01	0.957	1.020
Improvement in Verbal Recall (SRT)	97.490	9	<0.001	0.305	Age	–0.028	–0.28	3.909	1	0.048	0.972	0.946	1.000
					Education	0.033	0.040	0.681	1	0.409	1.033	0.956	1.116
					EMQ-p	–0.004	0.006	0.386	1	0.534	0.996	0.985	1.008
					SRT-s	–0.101	0.012	65.833	1	<0.001	0.904	0.882	0.926
					SDMT	0.021	0.012	2.823	1	0.093	1.021	0.997	1.046
					S10/36	0.020	0.028	0.509	1	0.476	1.020	0.965	1.079
					Doors and people								
					Oass	0.185	0.068	7.330	1	0.007	1.203	1.052	1.376
					Vrecalls	0.087	0.065	1.762	1	0.184	1.091	0.960	1.240
					vts	–0.083	0.074	1.244	1	0.265	0.921	0.796	1.065
					Constant	2.304	1.250	3.394	1	0.065	10.011		

SE: standard error; CI: confidence interval; EMQ-p: Everyday Memory Questionnaire-Participant; MSIS-Psy: Multiple Sclerosis Impact Scale-Psychological; RRMS: relapsing-remitting multiple sclerosis; PPMS: primary-progressive multiple sclerosis; MS: multiple sclerosis; PASAT2: Paced Auditory Serial Addition Test – 2 second; SRT-s: Selective Reminding Test – Long-Term Storage; S10/36: 10/36 Spatial Recall Test; Oass: Overall Age Scaled Score; Vrecalls: Verbal recall; Nvrecalls: Non-Verbal Recall Score; Nvrcogs: Non-Verbal Recognition Score; Nvts: Non-Verbal Total Score; Vts: Verbal Total Score; Nvts: Non-Verbal Total Score.

* Relapses refer to relapses that were experienced in the 6 months prior to receiving memory rehabilitation.

The odds of improvement decreased as participants’ baseline scores in the Everyday Memory Questionnaire (EMQ-*p*) increased (*p* < 0.001), suggesting that those who scored in the mild to moderate range of the questionnaire are more likely to benefit from cognitive rehabilitation than those who score severe. When mild, moderate, and severe groups were added into the model separately, both mild and moderate groups predicted improvement when compared to the severe group, confirming these results. However, this post hoc analysis was not significant.

The odds of improvement decreased as participants’ baseline scores on the Doors and People test increased (*p* < .001). During post hoc analysis, we found that participants that scored mid-range (5–8) in their total forgetting scores were more likely to improve compared to those scoring high (9–13) and low (0–4). This supports the finding that those with mild to moderate cognitive problems are most likely to benefit from cognitive rehabilitation.

### Psychological well-being

The final model (model *χ*^2^(6) = 108.523, *p* < 0.001) predicting improvement in psychological well-being following cognitive rehabilitation included type of MS (*p* = 0.042), relapses experienced in the 6 months prior to receiving the cognitive rehabilitation (*p* = 0.086) and baseline Multiple Sclerosis Impact Scale (MSIS-Psy)^17^ scores (*p* = 0.005). The odds of improvement were 1.016 times greater for people with relapsing-remitting multiple sclerosis (RRMS; *p* = .972), when compared to those with secondary-progressive multiple sclerosis (SPMS). A Nagelkerke’s pseudo *R*^2^ of .513 indicates a moderate explanatory power of the model. The final model correctly classified 86.6% of participants. The odds of improvement in psychological well-being were 2.139 times higher for those who had experienced relapses in the 6 months prior to receiving cognitive rehabilitation (*p* = 0.086).

### Sustained attention and processing speed

The final model (model *χ*^2^(12) = 70.322, *p* < 0.001) predicting improvement in the sustained attention and processing speed scores between baseline and 6 month follow-up following cognitive rehabilitation included younger age (*p* < 0.001), higher education (*p* = 0.137), higher baseline MSIS-Psy scores (*p* = 0.011), type of MS (*p* = 0.011), mild to moderate baseline Symbol Digit Modalities Test (SDMT) scores (*p* < 0.001) and mild to moderate baseline verbal recall scores (*p* < 0.021). A Nagelkerke’s pseudo *R*^2^ of 0.391 indicates a low to moderate explanatory power of the model. The final model correctly classified 80.9% of participants.

For type of MS, the odds of improvement were 2.617 times higher for people with RRMS (*p* = 0.039) and 2.237 times higher for people with primary-progressive multiple sclerosis (PPMS) (*p* = 0.278), compared to people with SPMS. The odds of improvement over time increased by 1.094 times per year of education. The odds of improvement decreased by .917 per year of age, suggesting that younger participants were more likely to benefit from cognitive rehabilitation.

### Attention, vigilance, and working memory

The final model (model *χ*^2^(14) = 76.847, *p* < 0.001) predicting improvement in attention, vigilance, and working memory following cognitive rehabilitation included younger age (*p* = 0.990), higher education (*p* = 0.815), baseline EMQ-*p* scores (*p* = 0.321), baseline Doors and People test scores (*p* = 0.003) and mild to moderate baseline PASAT-2 scores (*p* < 0.001). A Nagelkerke’s pseudo *R*^2^ of 0.420 indicates a moderate explanatory power of the model. The final model correctly classified 79.3% of participants.

For the Doors and People baseline scores, the odds of improvement decreased by 0.338 and 0.273 per point scored on the non-verbal recall (*p* = 0.008) and recognition subtests (*p* = 0.003), respectively. The odds of improvement decreased by 0.990 per year of age and increased by 1.014 per year of education; however, this was not significant. The chances of a participant showing improvement in attention, vigilance, and working memory following cognitive rehabilitation were reduced for those who had experienced one or more relapses 6 months prior to receiving the cognitive rehabilitation, however, this was not significant.

### Verbal memory and recall

The final model (model *χ*^2^(9) = 97.490, *p* < 0.001) predicting improvement in verbal memory and recall between baseline and 6 month follow-up following cognitive rehabilitation included younger age (*p* = 0.048), higher education (*p* = 0.409), baseline Selective Reminding Test (SRT-s) scores (*p* < .001), mild to moderate baseline SDMT scores (*p* = .093), mild to moderate baseline s10/36 scores (*p* = .476), and baseline doors and people scores (*p* = .007). A Nagelkerke’s pseudo *R*^2^ of 0.305 suggests low to moderate explanatory power of the model. The model correctly classified 71.2% of participants as showing improvement in verbal memory following group-based cognitive rehabilitation.

The odds of improvement over time decreased by 0.972 per year of age, suggesting that younger participants were more likely to improve their verbal memory following cognitive rehabilitation. The odds of improvement decreased by 0.904 per point scores on the SRT, suggesting that participants who scored lower were more likely to improve. The odds of improvement increased by 1.203 per point scores on the overall-aged score subset of the Doors and People test.

## Discussion

This study investigated the participant characteristics that predicted neuropsychological improvement in people with MS 6 months after 10 weeks of cognitive rehabilitation. Almost 80% of the intervention group improved in at least one of the six neuropsychological tests domains measured by both subjective measures of cognition and neuropsychological tests. Four categories of predictors of improvement in neuropsychological test performance were examined at the univariate level and, subsequently, in the context of a hierarchical logistical regression model.

Of all of the variables examined, the most frequently occurring significant predictors of neuropsychological improvement were (1) age, from the sociodemographic category; (2) type of MS and relapses experienced in the 6 months prior to receiving the cognitive rehabilitation, from the clinical category; (3) baseline everyday memory scores and psychological well-being scores, from the self-report cognitive symptoms category; and (4) baselines scores on the Doors and People Test, PASAT scores, and Selective Reminding Test scores, from the neuropsychological test category.

Participants with RRMS were more likely to report improvement of their psychological well-being, attention, vigilance, and working memory and sustained attention and processing speed over time when compared to those with SPMS. Participants with PPMS were less likely to report improvement in their psychological well-being but were more likely to report improvement in attention, vigilance, and working memory and sustained attention and processing speed when compared to those with SPMS. Participants who experienced a relapse in the 6 months prior to receiving cognitive rehabilitation were less likely to improve in terms of their cognition, but they were more likely to show improvement in their psychological well-being. This could be because their psychological well-being was reduced due to their recent relapse, and as the relapse symptoms presumably improved, the participant noticed some subsequent improvement of their psychological well-being. In outcome studies of patients with acquired brain injuries (without interventions specifically aimed at treating cognitive deficits), medical injury severity has been predictive of functioning.^18,19^ Cognitively, relapses might have caused further damage to the areas of the brain responsible for cognitive processes tested. There is evidence that cognition is notably more impaired during and immediately after an active relapse in people with MS compared to stable patients,^20^ so it is possible that patients immediately following a relapse may be slower to improve. A recent review posited that cognition declines acutely during a relapse and that cognitive impairment in people with MS may result from incomplete recovery of these relapses.^21^ However, there is a lot we still do not understand about the neuropathology driving cognitive relapse and recovery; thus, these results need to be interpreted with caution.

We found that younger participants were more likely to improve compared to older participants. This could be due to the nature of the cognitive rehabilitation programme. A significant part of this programme is teaching and encouraging participants to use external aids to help manage their cognitive difficulties. While a range of external aids are discussed, many focus on technology such as smartphones, mobile apps, smart home devices (e.g. Alexa), and online shared calendars. Using this technology may have come more naturally to those of a younger age; therefore, this may have contributed to the improvement in subjective measures of memory.

It is possible that age may play a significant role in predicting cognitive resilience or improvement linked to cognitive reserve.^22^ Higher levels of cognitive reserve in people with MS can protect against disease-related cognitive decline, and younger adults tend to have higher cognitive reserves compared to older adults.^23^ Studies have suggested that age can not only influence initial cognitive abilities but may also play a moderating role in the improvement of cognition during rehabilitation.^24,25^ Langbaum et al.^24^ investigated how healthy adults responded to memory training and concluded that higher education and higher baseline cognitive functioning were predictors of cognitive improvement, with age providing a moderating effect.

Age at diagnosis may also predict cognitive improvement with recent evidence suggesting cognitive impairment is more severe for people with MS who are diagnosed earlier.^26,27^ As a progressive disease, MS symptoms experienced gradually accumulate and worsen over time. Disease duration has been found to have a negative impact on both processing speed and working memory.^28^

Although education was repeatedly found to be a notable factor in predicting improvement in several of the models, it did not contribute significantly. The literature suggests that higher education is predictive of improvement or faster recovery of cognition in older healthy adults and could be protective against MS associated cognitive deficits.^24^ Furthermore, the cognitive rehabilitation programme teaches the use of internal aids such as mnemonic strategies (e.g. visualisation, categorisation, chunking, repetition, etc.), and the ability to self-generate these mnemonic strategies is associated with higher education, which could lead to improved memory and better performance on memory tasks.^29,30^

Education may be a predictor of improvement in cognition mediated possibly through employment. Highly educated people with MS being more likely to remain in work compared to those with a lower education.^31^ Being employed may make people with MS more motivated to engage in cognitive rehabilitation effectively, which could influence performance on the cognitive tests performed after cognitive rehabilitation.

In terms of socio-demographics, we found that people with MS living with others were more likely to experience improvement in their memory than those living alone. This could be explained as the partner or family might be able to provide emotional support to the participants during the intervention period as well as helping with some of the technical aspects of using external memory aids, as it is well supported that having an adequate support system can positively impact on patient outcomes (refs).^32^

Baseline neuropsychological tests, such as lower verbal recall and recognition test scores, were a significant factor in predicting improvement. Although no participants were performing at ceiling and therefore, all participants had room for improvement, it is plausible that those who scored lower simply had more room for improvement. Post hoc analysis provided some support for this explanation as, when we examined the mean gain improvement scores in those that scored below the mean on the EMQ-p at baseline, greater improvements were seen compared to those that score above the mean.

In summary, our findings suggest younger patients with medium to high education, diagnosed with RRMS and PPMS without recent relapses, living with a partner or other family, with mild to moderate cognitive difficulties are more likely to benefit from memory rehabilitation than other groups.

One of the strengths of this study owing to the large sample size of CRAMMS, is that we were able to consider several predictor and outcome variables. Of course, the main limitation of this post hoc analysis is that our findings are based on retrospective data. Therefore, findings from this study should be confirmed using a prospective research design, which we are currently undertaking. Furthermore, as this is a post hoc analysis, some of the sample sizes for individual groups such as participants who were diagnosed with the less common types of MS (e.g. PPMS) were small. As very few neuropsychological tests have clinically meaningful definitions of improvement, we chose to define improvement as any positive change in scores between baseline and the 6-month follow-up, which could be considered a limitation of this study.

Improvement in some cognitive domains is more predictable than improvement in others. For instance, predicting improvement in subjective measures of memory and psychological well-being appear to be more accurate and accounts for greater variance than predicting improvement in sustained attention and processing speed or verbal memory. This could be due to the mechanism driving improvement in cognition being more complex for neuropsychological tests. We also must note that the models vary in the amount of variance associated with improvement that they account for. However, the final models were able to correctly predict between 71.2% and 86.6% whether participants improved or did not improve following cognitive rehabilitation. This has significant clinical implications as one of the key reasons cognitive rehabilitation programmes are not routinely offered is due to there not being the resources and staff to offer it to everyone with MS. However, if we can identify those that are more likely to benefit, and the results of this study suggest that we may be able to do just that, then we can target the cognitive rehabilitation to those most likely to benefit and design (and offer) other interventions for those who are less likely to benefit.
